# A Note on the Association Between Climatological Conditions and the Presence of *Coxiella burnetii* in the Milk-Tank of Dairy Sheep and Goat Farms in Greece

**DOI:** 10.3390/pathogens14070686

**Published:** 2025-07-12

**Authors:** Eleni I. Katsarou, Themistoklis Giannoulis, Charalambia K. Michael, Daphne T. Lianou, Natalia G. C. Vasileiou, Nikolaos Solomakos, Angeliki I. Katsafadou, Vasia S. Mavrogianni, Dimitriοs C. Chatzopoulos, George C. Fthenakis

**Affiliations:** 1 Veterinary Faculty, University of Thessaly, 43100 Karditsa, Greece; elekatsarou@vet.uth.gr (E.I.K.); dlianou@vet.uth.gr (D.T.L.); nsolom@uth.gr (N.S.); vmavrog@vet.uth.gr (V.S.M.); 2 Faculty of Animal Science, University of Thessaly, 41110 Larissa, Greece; thgianno@uth.gr (T.G.);; 3 School of Veterinary Medicine, European University of Cyprus, Engomi, 2404 Nicosia, Cyprus; cha.michael@euc.ac.cy (C.K.M.); dchatzopoulos@uth.gr (D.C.C.); 4 Faculty of Public and One Health, University of Thessaly, 43100 Karditsa, Greece; agkatsaf@vet.uth.gr

**Keywords:** predictors, climate, goat, sheep, *Coxiella burnetii*, Q fever, wind

## Abstract

The specific objectives of the current paper were the assessment of potential associations of weather conditions with the presence of *Coxiella burnetii* in the milk-tank of sheep and goat farms and the investigation for possible interactions between weather conditions and management practices on these farms. The presence of *C. burnetii* in milk-tank samples collected from 325 sheep flocks and 119 goat herds was assessed by means of a commercially available real-time PCR. Climatic variables present at the location of each farm were downloaded from ‘The POWER Project’. Univariable and multivariable analyses were carried out. Among the climatic variables assessed, only the average wind speed during the 15 days that preceded each visit was found to be a significant predictor for both sheep (*p* = 0.003) and goat (*p* = 0.034) farms. The current findings serve to provide information about the epidemiology of *C. burnetii* infections in small ruminant farms and the possibilities for contamination of the milk produced in these farms, which is important due to the zoonotic nature of the pathogen; these findings thus provide guidance to implement appropriate preventive measures.

## 1. Introduction

Various research works have highlighted the role of climate conditions in the development of infectious diseases, as climate can exert a range of direct and indirect influences on diseases. In general, climatic conditions can affect host defenses, as heat or water stress can impair the immune function of animals and humans [1]. Further, climate conditions can modulate the spatial distribution, seasonality, and development rate of disease-causing pathogens [2].

*Coxiella burnetii* is an established pathogen of ruminants, which in animals is responsible for reproductive disorders [3,4,5]. More importantly, *C. burnetii* is also a particularly significant zoonotic pathogen, the aetiological agent of Q fever, which usually is an asymptomatic or mild infection, with fatigue, fever, or respiratory signs [6,7]. This infectious disease is difficult to diagnose, first, due to the difficulty of discriminating it as the clinical signs are not characteristic, and, second, because the diagnostic tests often provide negative results [8,9,10].

Infection of ruminants and humans takes place often through the inhalation of aerosolized particles of the pathogen, which are dispersed in the environment, and in this way disseminate to infect susceptible individuals [11]. In small ruminant farms, infections of animals and workers can often result due to the pathogen’s dissemination within animal houses. For example, during the parturition of infected ewes or does, huge numbers of pathogen particles from the amniotic fluid of the females are dispersed within the environment of the farm and can infect animals within the same housing facility (which can lead to abortion of the previously uninfected animals) or people nearby, e.g., veterinarians or farmers, through the inhalation of the pathogen particles [11,12].

Further, the consumption of milk or dairy products containing the pathogen is another means through which human infections can be initiated [6]. In Greece, where the consumption of cheese from sheep or goat milk is frequent, a study into Q fever, performed some years ago, revealed that children who had consumed cheese made from small ruminant milk had a higher risk for infection and development of the disease [13].

Previous studies have reported the association of wind speed with the dissemination of *C. burnetii* among animals (ruminants) or between animals and people [14,15]. In fact, the pathogen may be dispersed as far as 18 km by means of the wind [16]. Moreover, an association has been reported between the weather conditions and the development of Q fever in people [17], whilst Bauer et al. [18] also reported that dry and windy weather conditions favoured the dissemination of the causal pathogen. More recently, Mosikidi et al. [19] reported a higher seroprevalence of *C. burnetii* infection in small ruminants during windy periods. However, the potential effects on the presence and detection of the organism in the milk-tank were not reported previously.

The specific objectives of the current paper were the assessment of potential associations of weather conditions with the presence of *C. burnetii* in the milk-tank of sheep and goat farms and the investigation for possible interactions between weather conditions and management practiced on these farms. There is interest in this evaluation, first because of the importance of dairy ovine and caprine milk production in Greece, which overall accounts for almost 1% of the total GDP of the country, as well as because of the changes in climate conditions, which thus may influence animal health in small ruminant farms. The present study follows on a recently published paper [20], where we have described the frequency of the presence of *C. burnetii* in the milk produced in the farms and also have explored the potential associations with management-related factors.

## 2. Materials and Methods

**Figure 1:**
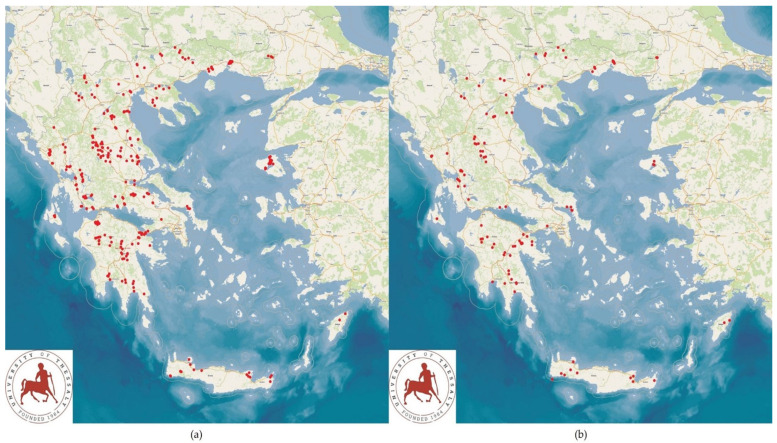
Locations of the farms throughout Greece, wherein the presence of *C. burnetii* genetic material was investigated ((**a**): sheep farms, (**b**): goat farms) (red dots denote locations of farms into the study).

The study was carried out in 444 small ruminant farms (325 sheep flocks and 119 goat herds; 222 farms with intensive or semi-intensive management and 222 farms with semi-extensive or extensive management) located in Greece, throughout the country in all the 13 administrative regions (Figure 1). The work was performed from April 2019 to July 2020. Farms were selected on a convenience basis and included those that would accept a visit by university personnel for the collection of data and samples. The visits were carried out in association with veterinarians, who made the arrangements with the farmers and also accompanied the researchers during the visits [20]. Visits to farms were made during all four seasons of the year, during the spring (*n* = 151), the summer (*n* = 141), the autumn (*n* = 41), and the winter (*n* = 111).

Visits were performed at all these farms and samples were collected from the milk-tank therein, as described previously [20]. On each farm, during the visit, four 20 mL samples were collected directly from the milk-tank, by means of sterile single-use pipettes, whilst maintaining full aseptic conditions during the sampling.

After the transport of samples to the laboratory, initially, DNA extraction was performed on the samples from the milk-tank collected from each farm (IndiSpin Pathogen Kit; Indical BioScience, Leipzig, Germany). The detection of the DNA of the pathogen followed and was performed by employing a commercially available qPCR kit (bactotype *C. burnetii* PCR Kit; Indical BioScience, Leipzig, Germany), by following the protocol of the manufacturer [20]. The conditions for the qPCR conditions were the following: first denaturation step for 5 min at 95 °C, second denaturation step (40 cycles) for 10 s at 95 °C, and, finally, an annealing/extension step for 30 s at 57 °C [20].

During the visit to each farm, data about the geo-location of each farm were obtained by means of Global Positioning System Garmin units. The geo-references were resolved to the specific farm level.

Details about the climatic variables that prevailed at the location of each farm in relation to the time of sample collection there, were obtained from the platform of ‘The POWER (Prediction of Worldwide Energy Resources) Project’ (NASA Langley Research Center (LaRC), Hampton, VA, USA). This facility collects datasets regarding meteorological conditions and parameters, obtained from NASA research, and makes these available specifically to support agricultural needs and relevant scientific work. The settings employed for obtaining the data were as follows: ‘agroclimatology’, ‘daily & annual’, ‘geo-references of each farm’, ‘ASCII’. Data for the following eight (8) climatic parameters were extracted: ‘temperature at 2 m’ (i.e., at a height of 2 m above the surface), ‘temperature of Earth skin’, ‘minimum temperature at 2 m’, ‘maximum temperature at 2 m’, ‘temperature range at 2 m’, ‘relative humidity at 2 m’, ‘total precipitation’, and ‘wind speed at 10 m’ [21]. For the evaluation of the significance of weather-related variables, the above weather parameters for the seven (7) and fifteen (15) days before each visit were considered.

The associations of the presence of the genetic material of the pathogen in the milk collected from farms with each of 16 climate-related parameters were evaluated initially in univariable analyses by means of Spearman’s rank correlation analysis. This was followed with multivariable analysis, which was performed using mixed-effects logistic regression with the farms as the random effect, after inclusion into the model of all variables that had achieved a significance of *p* < 0.25 in the earlier univariable analysis; this was followed by progressive removal of variables [20]. Separate analyses were performed for sheep and goat farms (Table S1).

Subsequently, possible interactions between weather conditions and management were investigated in farms with intensive or semi-intensive (I/s-I) management, in which the presence of the genetic material of the pathogen was high (5.9%) and in farms with semi-extensive or extensive (s-E/E) management, in which the presence of the genetic material of the pathogen was low (0.9%) [20]. For this analysis, the following variables were taken into consideration: first, all climatic variables into the respective final models (*n* = 3 for sheep farms and *n* = 1 for goat farms; Table S1), and, second, all management variables into the final models of the relevant study previously performed (*n* = 5 for sheep farms, specifically the following: availability of accessory building(s) for animals, availability of a dedicated building for young animals, average age of culling female animals, seasonal transfer of animals to another site, provision of silage to adult animals; and *n* = 2 for goat farms, specifically the following: presence of pigs on the farm, presence of equines on the farm [20]). Again, separate analyses were performed for sheep and goat farms (Table S1). Thereafter, classification analysis was carried out between the variables included in the final model for the detection of the pathogen in sheep or goat farms under I/s-I management (Table S1); for this, the wind speed was transformed to a qualitative characteristic and farms were allocated into classes by taking into account the quartile among all wind speed data, into which each farm was allocated: ‘1’ for the lower quartile, ‘2’ for the second quartile, ‘3’ for the third quartile, and ‘4’ for the upper quartile.

For the characterization of the type of management applied on each farm (‘intensive’, ‘semi-intensive’, ‘semi-extensive’, ‘extensive’), the classification published by the European Food Safety Authority [22] was adopted and used. Statistical significance was defined at *p* < 0.05.

## 3. Results

In all, genetic material of *C. burnetii* was detected in samples from fifteen (15) farms (3.4%; 95% confidence interval (CI): 2.1–5.5%). There was no difference between this proportion among sheep (2.8%; 95% CI: 1.5–5.2%) or goat (5.0%; 95% CI: 2.3–10.6%) farms (*p* = 0.24). However, this proportion was significantly higher among farms with I/s-I management (5.9%; 95% CI: 3.5–9.8%) than among farms with s-E/E management (0.9%, 95% CI: 0.3–3.2%) (*p* = 0.004).

The findings of the univariable analyses are shown in Table S2. Wind speed for the 15 days before the visit and sampling was significantly faster at the locations of farms where *C. burnetii* was detected than in farms where it was not detected. Similar results were found for sheep and goat farms alike: the relevant median values of wind speed among the respective farms were 2.69 (interquartile range (IQR): 5.78) m s^−1^ and 2.96 (IQR: 3.32) m s^−1^ versus 2.12 (IQR: 2.70) m s^−1^ and 2.18 (IQR: 2.52) m s^−1^, respectively (*p* = 0.06 for sheep farms, *p* = 0.017 for goat farms).

**Figure 2:**
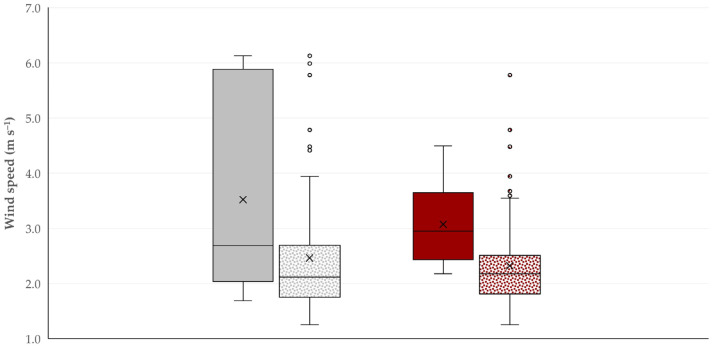
Box and whisker plot of the average wind speed for the 15 days before the visit and sampling, at the locations of sheep farms (gray) or goat farms (brown) where *C. burnetii* genetic material was (full bars) or was not (motif bars) found in milk samples from the milk-tank. Legend: x: mean value of data; bar within the box: median value of data; upper and lower limits of the box: lower and upper quartile of data; difference between lower and upper limits of the box: interquartile range; lower and upper ‘whiskers’: lower and upper extremes of data excluding outliers; dots above the upper whiskers: upper outliers [23].

During the multivariable analysis, only the average wind speed at the locations of farms for the 15 days that preceded each visit and sampling was found to be a significant predictor for both sheep farms (*p* = 0.003) and goat farms (*p* = 0.034) (Table 1, Figure 2).

**Figure 3:**
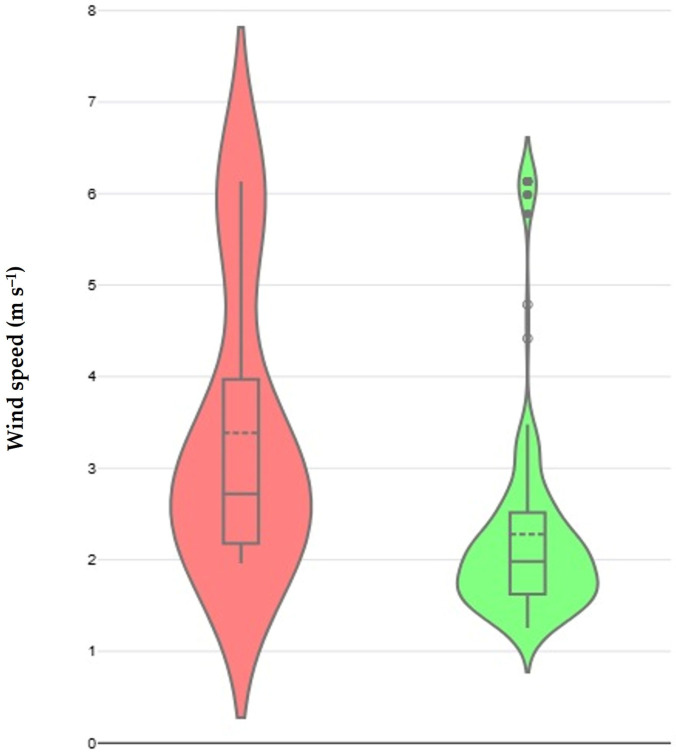
Violin plot of the wind speed for the 15 days before the visit and sampling, at the locations of small ruminant farms under intensive or semi-intensive management where *C. burnetii* genetic material was (red) or was not (green) found in samples from the milk-tank.

During the multivariable analysis among the farms under I/s-I management, again, only the average wind speed at the locations of farms for the 15 days that preceded each visit and sampling emerged as a significant variable both for sheep (*p* = 0.006) and goat (*p* < 0.0001) farms (Table 2, Figure 3). Moreover, specifically for sheep farms, a tendency for association was found for the availability on the farm of accessory building(s) for animals (*p* = 0.07). For the farms where s-E/E management system was applied, no significant association was found between the detection of *C. burnetii* in the milk-tank and the climatic variables assessed in the univariable analyses performed, in both sheep (*p* > 0.23 for all evaluations) and goat (*p* > 0.22 for all evaluations) farms.

**Figure 4:**
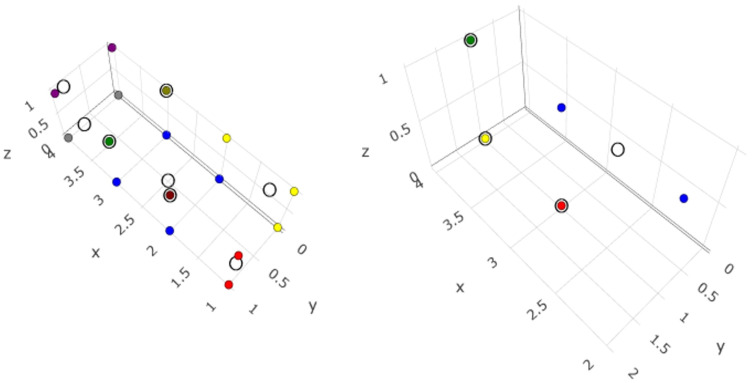
Visualization of analysis of combinations of potential predictors for the detection of *C. burnetii* in the milk-tank of sheep farms under intensive/semi-intensive management; left graph: farms in which *C. burnetii* was not detected (91.5% of variance could be explained with eight clusters)—right graph: farms in which *C. burnetii* was detected (91.7% of variance could be explained with four clusters).

**Figure 5:**
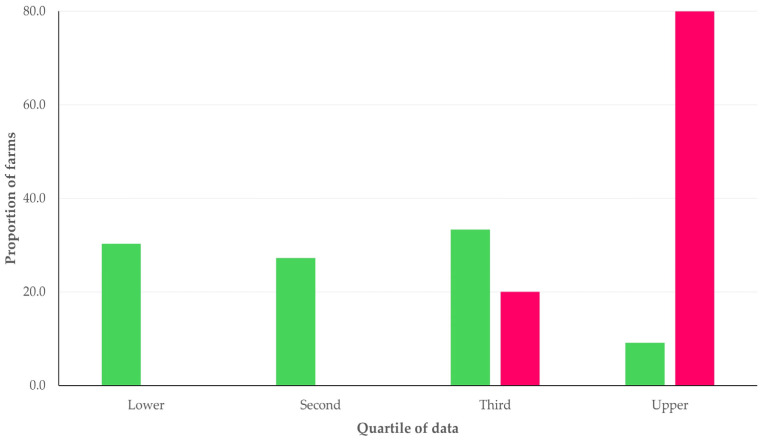
Distribution of goat farms under intensive/semi-intensive management among the four quartiles of the data regarding wind speed at 10 m 15 d prior to the visit and sampling, in accordance with detection (red bars) or no detection (green bars) of *C. burnetii* in the milk-tank.

In the classification analysis, it became evident that the most frequently encountered clusters for sheep farms under I/s-I management without *C. burnetii* detection were the following: (a) second quartile for wind speed data, availability of accessory building(s), availability of a dedicated building for lambs, which included 18.8% of farms (*n* = 33) and (b) third quartile for wind speed data, availability of accessory building(s), availability of a dedicated building for lambs, and no seasonal transfer of animals, which included 17.0% of farms (*n* = 30). In contrast, the most frequently encountered clusters for farms where *C. burnetii* was detected were the following: (a) upper quartile for wind speed data, no availability of accessory building(s), availability of a dedicated building for lambs, which included 37.5% of farms (*n* = 3) and (b) upper quartile for wind speed data, availability of accessory building(s), availability of a dedicated building for lambs, which included 25.0% of farms (*n* = 2) (Table S3). In goat farms under I/s-I management, the most frequently encountered clusters were the third quartile (33.3% of farms, *n* = 11) and the lower quartile (30.3% of farms, *n* = 10) for farms where *C. burnetii* was not detected, and the upper quartile (80.0% of farms, *n* = 4) for farms where *C. burnetii* was detected (Table S3) (Figure 4 and Figure 5).

Finally, it is noted that among the fifteen farms in which the pathogen was detected in the milk-tank, two were located less than 1.5 km apart. Among the other thirteen farms, in two pairs of farms the distance between the two farms in the same pair was 30 to 35 km, whilst all other distances between farms were over 50 km.

## 4. Discussion

The present findings may first indicate a contamination of the milk-tank with *C. burnetii*, which may take place in cases of high wind speed prevailing at farm locations, if the milk-tank would not be correctly closed and secured [11,24]. Another possibility for the presence of *C. burnetii* in the milk-tank can be the excretion of the pathogen in the milk produced by the farm animals at the time of the visit, which had been infected earlier. Tissot-Dupont et al. [25] reported an outbreak of Q fever in sheep one month after the sudden development of gusty winds in sheep farms near the coastal city of Marseilles, France.

Apart from the increased dissemination of the pathogen, as the direct effect of the wind speed, one should take into account that adverse weather effects may have more general consequences on the immunity of the animals [26]. This can lead to higher pathogen numbers infecting the animals, ultimately leading to the shedding of higher *Coxiella* loads in the milk. In such cases, the correct nutritional management of animals under stress related to climate variables can help to minimize the possible adverse effects [27]. Wind and wind speed are closely linked with atmospheric pressure, as winds occur as the result of horizontal and vertical differences in pressure. The concentration of glucocorticoids, which have an established association with the immune response of animals, in the blood of animals could increase with changes in atmospheric pressure [28], which also leads to winds. This provides a potential link between winds and wind speed and the response of animals to pathogens under such weather conditions, which may trigger the development of various disorders, including more severe infections [29].

Another possible alternative, although with only a small possibility of occurrence, may be the infection of animals through ticks carried by the wind into a farm, especially if the ticks were attached to objects (e.g., leaves or small pieces of vegetation) blown by the wind [30]. England et al. [31] reported that wind and static electricity can facilitate the attachment of ticks to new hosts, which may lead to infection of the animals and transmission of the pathogen. Finally, another possibility could be the contamination of the milk-tank by people working on the farm (farmer, milkers) either at the time of milking of the animals or when handling the milk-tank.

In a subsequent analysis, we focused on evaluating predictors for farms managed under I/s-I management, as the significant majority of cases of detection of the pathogen occurred in such farms. The results indicated that to some extent, management factors might be involved. To some extent, their involvement may also be associated with the increased wind speed found as a significant predictor. For example, the housing of sheep during windy conditions has been reported to contribute to improved performance of the animals, as this can provide a better microenvironment for the animals [32]; however, the housing of animals can contribute to the dissemination of the pathogen between animals, which is subsequently excreted in their milk.

Wind speed clearly contributes to the dissemination of the pathogen. Its particular significance among farms where intensive/semi-intensive management is applied, along with the tendency for the significance of variables related to infrastructure on the farms, suggests that, although dissemination of the pathogen is facilitated by the wind, as discussed above, the management system applied on the farms also contributes therein.

The identification of a pair of farms in close proximity, in both of which the pathogen was detected, indicates a potential for dissemination of the pathogen between these two farms by means of the wind. In rural areas, the maximum risk for infection occurs in farms within a distance of 5 km [15], although during gale winds, the dispersal of the pathogen for up to 18 km has been reported [15]. This also underlines the necessity for sharing information between farms as part of the efforts to minimize the transmission of pathogens between them.

Nevertheless, high wind speed at a farm location cannot be prevented, but it can nevertheless be monitored, with the aim of reducing the dissemination of the pathogen. That way, the potential adverse consequences of high wind speed can be minimized. Hence, during windy weather conditions, farmers should take increased precautions to prevent the possible contamination of their milk-tank, as well as to minimize the possible dispersal of the pathogen, particularly after the parturition of animals. Moreover, dairy industries should be alert to pay greater attention to processing the milk brought into plants subsequently to windy weather.

Further, one may suggest that on farms at locations where increased wind speeds prevail (for example, on the islands of the country [21]), farmers should apply greater and more consistent vigilance, including taking specific health management measures to prevent the incursion of the pathogen. *C. burnetii* can survive in the dust and the environment in general for extended periods. During dry weather, the contaminated particles become aerosolized and in cases of increased wind they are dispersed and may infect people and animals. Notably also, Zeng et al. [33] have reported an upsurge in wind speeds over land since the year 2010. This trend may lead to the increased dissemination of the pathogen in the future, which also calls for implementing relevant preventive measures, in order to minimize the possible dissemination of the pathogen into the milk produced in small ruminant farms. In turn, this will lower the risk of the dissemination of the pathogen to dairy products and its possible transmission to consumers.

The current findings serve to provide information about the epidemiology of *C. burnetii* infection in small ruminant farms and the possibilities for the contamination of the milk produced in these farms, which is important due to the zoonotic nature of the pathogen; these findings thus provide guidance to implement appropriate preventive measures. Therefore, when carrying out the various tasks routinely performed on farms (for example, lambing routines, application of manure on the farm land), one should always account for the possibility of pathogen dispersal by means of the wind. That way, relevant preventive measures can be enforced; among others, such measures can include the management of parturient ewes/does in an area with controlled airflow, the transfer of pregnant animals to upwind parts of the farms, and the reduction of the dusty environments within and around the housing facilities in the farms. Moreover, and in view of the above, the wind speed can also be incorporated into indices related to the assessment of the effects of climate-related variables on animals. That way, such indices can be used to improve the health and welfare of animals [34].

## Figures and Tables

**Table 1:** Findings of multivariable analysis for climate-related variables associated with the presence of *C. burnetii* in samples from the milk-tank of small ruminant farms.

Variables	Odds Risk (±se)	*p*
Sheep Farms		
Average wind speed for the 15 days before the visit		0.003
Increase per unit (m s^−1^)	1.02 ± 1.01	0.007
Goat Farms		
Average wind speed for the 15 days before the visit		0.034
Increase per unit (m s^−1^)	1.05 ± 1.02	0.034

**Table 2:** Findings of multivariable analysis for climate- and management-related variables associated with the presence of *C. burnetii* in samples from the milk-tank of small ruminant farms under intensive or semi-intensive management.

Variables	Odds Risk (±se)	*p*
Sheep Farms		
Average wind speed for the 15 days before the visit		0.004
Increase per unit (m s^−1^)	1.04 ± 1.01	0.002
Goat Farms		
Average wind speed for the 15 days before the visit		<0.0001
Increase per unit (m s^−1^)	1.46 ± 1.09	<0.0001

## Data Availability

Data presented in this study are contained within the text.

## Supplementary Materials

The following supporting information can be downloaded at: https://www.mdpi.com/article/10.3390/pathogens14070686/s1, Table S1: Details of multivariable models employed for the evaluation of potential associations of climatic variables with the detection of genetic material of *Coxiella burnetii* in the bulk-tank milk of 444 small ruminant farms; Table S2: Results of univariable analysis for the detection of genetic material of *Coxiella burnetii* in the milk-tank of 444 small ruminant farms; Table S3: Results of classification analysis for potential predictors for the detection of genetic material of *Coxiella burnetii* in the milk-tank of 222 small ruminant farms under intensive or semi-intensive management.
