# Supplementary material for: A Note on the Association Between Climatological Conditions and the Presence of Coxiella burnetii in the Milk-Tank of Dairy Sheep and Goat Farms in Greece
Source: Pathogens. 2025 Jul 12;14(7):686. doi: 10.3390/pathogens14070686 (PMC12300821; doi:10.3390/pathogens14070686)
Supplement: Supplementary file 1 [file pathogens-14-00686-s001.zip › pathogens-3699445-supplementary.pdf]

A note on the Association Between Climatological Conditions and the Presence of *Coxiella burnetii* in the Milk-Tank of Dairy Sheep and Goat Farms in Greece

E.I. Katsarou, T. Giannoulis, C.K. Michael, D.T. Lianou [1], N.G.C. Vasileiou, N. Solomakos, A.I. Katsafadou, V.S. Mavrogianni, D.C. Chatzopoulos, and G.C. Fthenakis

Table S1. Details of multivariable models employed for the evaluation of potential associations of climatic variables with the detection of genetic material of *Coxiella burnetii* in the milk-tank of 444 small ruminant farms.

utcome	Variables		
	**assessed in univariable analyses (*n*)**	**offered to the multivariable models (*n*)**	**required in the final models**
Detection of genetic material of *C. burnetii* in the milk-tank from all sheep farms	16	5	(a) minimum temperature at 2 m 15 d prior to sampling, (b) maximum temperature at 2 m 15 d prior to sampling, (c) wind speed at 10 m 15 d prior to sampling
Detection of genetic material of *C. burnetii* in the milk-tank from all goat farms	16	5	(a) wind speed at 10 m 15 d prior to sampling

Outcome	Variables		
	**assessed in univariable analyses (*n*)**	**offered to the multivariable models (*n*)**	**required in the final models**
Detection of genetic material of *C. burnetii* in the milk-tank from sheep farms under intensive or semi-intensive management	--	8	(a) availability of accessory building(s) for animals, (b) availability of a dedicated building for lambs, (c) wind speed at 10 m 15 d prior to sampling
Detection of genetic material of *C. burnetii* in the milk-tank from goat farms under intensive or semi-intensive management	--	3	(a) wind speed at 10 m 15 d prior to sampling

Table S2. Results of univariable analysis for the detection of genetic material of *Coxiella burnetii* in the milk-tank of 444 small ruminant farms.

(a) Sheep flocks (n = 325)

Variables	r_{sp}	p value
temperature at 2 m for 7 days prior to sampling	0.018	0.75
temperature of Earth skin for 7 days prior to sampling	0.019	0.73
minimum temperature at 2 m for 7 days prior to sampling	0.022	0.69
maximum temperature at 2 m for 7 days prior to sampling	0.024	0.67
temperature range at 2 m for 7 days prior to sampling	–0.018	0.75
relative humidity at 2 m for 7 days prior to sampling	–0.018	0.75
total precipitation for 7 days prior to sampling	0.060	0.28
wind speed at 10 m for 7 days prior to sampling	–0.034	0.54
temperature at 2 m for 15 days prior to sampling	–0.074	0.18
temperature of Earth skin for 15 days prior to sampling	–0.087	0.12
minimum temperature at 2 m for 15 days prior to sampling	–0.073	0.19
maximum temperature at 2 m for 15 days prior to sampling	–0.073	0.19
temperature range at 2 m for 15 days prior to sampling	0.015	0.79
relative humidity at 2 m for 15 days prior to sampling	-0.063	0.26
total precipitation for 15 days prior to sampling	–0.053	0.34
wind speed at 10 m for 15 days prior to sampling	0.100	0.07

(b) Goat herds (n = 119)

Variables	r_{sp}	p value
temperature at 2 m for 7 days prior to sampling	0.097	0.29
temperature of Earth skin for 7 days prior to sampling	0.109	0.24
minimum temperature at 2 m for 7 days prior to sampling	0.100	0.28
maximum temperature at 2 m for 7 days prior to sampling	0.105	0.26
temperature range at 2 m for 7 days prior to sampling	–0.007	0.94
relative humidity at 2 m for 7 days prior to sampling	–0.115	0.21
total precipitation for 7 days prior to sampling	–0.068	0.46
wind speed at 10 m for 7 days prior to sampling	0.003	0.97
temperature at 2 m for 15 days prior to sampling	0.099	0.29
temperature of Earth skin for 15 days prior to sampling	0.099	0.29
minimum temperature at 2 m for 15 days prior to sampling	0.113	0.22
maximum temperature at 2 m for 15 days prior to sampling	0.064	0.49
temperature range at 2 m for 15 days prior to sampling	–0.203	0.027
relative humidity at 2 m for 15 days prior to sampling	0.026	0.78
total precipitation for 15 days prior to sampling	–0.082	0.38
wind speed at 10 m for 15 days prior to sampling	0.228	0.013

Table S3. Results of classification analysis for potential predictors for the detection of genetic material of *Coxiella burnetii* in the milk-tank of 222 small ruminant farms under intensive or semi-intensive management.

(a) Sheep flocks (*n* = 184)

Variables			No. of farms	
Wind speed at 10 m 15 d prior to sampling	Availability of a dedicated building for lambs	Availability of accessory build-ing(s) for animals	Without detection of *C. burnetii* [1]	With detection of *C. burnetii* [1]
Lower quartile	No	No	6 (3.4%)	0 (0.0%)
Lower quartile	No	Yes	7 (4.0%)	0 (0.0%)
Lower quartile	Yes	No	8 (4.5%)	0 (0.0%)
Lower quartile	Yes	Yes	26 (14.8%)	0 (0.0%)
Second quartile	No	No	3 (1.7%)	0 (0.0%)
Second quartile	No	Yes	5 (2.8%)	0 (0.0%)
Second quartile	Yes	No	3 (1.7%)	0 (0.0%)
Second quartile	Yes	Yes	33 (18.8%)	1 (12.5%)
Third quartile	No	No	3 (1.7%)	0 (0.0%)
Third quartile	No	Yes	8 (4.5%)	0 (0.0%)
Third quartile	Yes	No	3 (1.7%)	1 (12.5%)
Third quartile	Yes	Yes	30 (17.0%)	1 (12.5%)
Upper quartile	No	No	4 (2.3%)	0 (0.0%)
Upper quartile	No	Yes	4 (2.3%)	0 (0.0%)
Upper quartile	Yes	No	9 (5.1%)	3 (37.5%)
Upper quartile	Yes	Yes	24 (13.7%)	2 (25.0%)

1: percentages in brackets indicate proportion of farms among all those in the respective column.

(b) Goat herds (*n* = 38)

Variables	No. of farms	
Wind speed at 10 m 15 d prior to sampling	Without detection of *C. burnetii* [1]	With detection of *C. burnetii* [1]
Lower quartile	10 (30.3%)	0 (0.0%)
Second quartile	9 (27.3%)	0 (0.0%)
Third quartile	11 (33.3%)	1 (20.0%
Upper quartile	3 (9.1%)	4 (80.0%)

1: percentages in brackets indicate proportion of farms among all those in the respective column.
